# IRE1α modulates M1 oncolytic virus sensitivity via ER stress regulation in bladder cancer

**DOI:** 10.20517/cdr.2025.119

**Published:** 2025-08-13

**Authors:** Cheng Hu, Song Wei, Wenbo Zhu, Boran Lv, Shuhao Li, Baiyu Liu, Guangmei Yan, Ying Liu

**Affiliations:** ^1^Department of Urology, The Third Affiliated Hospital, Sun Yat-sen University, Guangzhou 510630, Guangdong, China.; ^2^Department of Pharmacology, Zhongshan School of Medicine, Sun Yat-sen University, Guangzhou 510080, Guangdong, China.; ^3^Department of Infectious Diseases, The Third Affiliated Hospital, Sun Yat-sen University, Guangzhou 510630, Guangdong, China.; ^#^Authors contributed equally.

**Keywords:** Oncolytic M1 virus, IRE1α, ER stress, UPR, bladder cancer

## Abstract

**Aim:** Muscle-invasive bladder cancer (MIBC) remains lethal despite promising oncolytic virotherapy, hindered by tumor-intrinsic resistance. This study aimed to elucidate the molecular basis underlying differential sensitivity to the oncolytic M1 virus in bladder cancer.

**Methods:** Bladder cancer cell lines with varying sensitivity to M1 were analyzed for endoplasmic reticulum (ER) stress responses and unfolded protein response (UPR) pathway activation. IRE1α expression was modulated using small interfering RNA and a selective inhibitor. Viral cytotoxicity, replication, and apoptosis were assessed using viability assays, immunofluorescence, electron microscopy, and immunoblotting. *In vivo* antitumor efficacy was assessed using xenografted mice. Clinical relevance was examined using patient-derived cells and survival data from The Cancer Genome Atlas.

**Results:** M1 virus induced ER stress and apoptosis in sensitive cells (e.g., T24, UM-UC-3) supporting viral protein expression, whereas low-sensitivity cells like EJ showed minimal response due to limited viral replication. In moderately sensitive cells, M1 replication led to viral protein accumulation, triggering IRE1α upregulation, which in turn limited further protein buildup and apoptosis. IRE1α inhibition enhanced M1-induced ER stress, apoptotic signaling, and oncolysis without affecting viral replication capacity. *In vivo*, M1 plus STF083010 achieved greater tumor suppression than monotherapy without added toxicity. Analysis of patient-derived cells and TCGA data further revealed downregulation of IRE1α in primary tumors and its potential association with worse prognosis.

**Conclusion:** IRE1α modulates M1-induced viral protein accumulation and cell death. Inhibiting IRE1α enhances ER stress and potentiates the oncolytic effect of M1 virus. Targeting IRE1α may improve M1-based virotherapy outcomes in accessible tumors.

## INTRODUCTION

Bladder cancer is a common malignancy of the urinary system and poses a considerable global health burden, with hundreds of thousands of new cases and substantial mortality reported annually^[1,2]^. Over 90% are urothelial carcinomas, categorized into non-muscle-invasive (NMIBC) and muscle-invasive bladder cancer (MIBC), with the former representing the majority of newly diagnosed cases^[3,4]^. Standard treatments include surgery, chemotherapy, immunotherapy, and intravesical therapy. For high-grade NMIBC, transurethral resection followed by Bacillus Calmette–Guérin (BCG) instillation remains the mainstay^[5,6]^. However, up to 50% of patients relapse, and 30% progress to MIBC despite BCG treatment^[7]^. MIBC often requires radical cystectomy, urinary diversion, and lymph node dissection, procedures associated with high morbidity, especially in elderly or comorbid patients^[8,9]^. For advanced or metastatic MIBC, systemic chemotherapy is commonly used, but staging inaccuracies and potential treatment delays limit its effectiveness, particularly in chemoresistant patients^[10]^. Patients progressing to MIBC after BCG failure generally have a poorer prognosis than those initially diagnosed with MIBC^[11]^. Although treatment modalities have improved, MIBC continues to exhibit a high rate of recurrence and poor long-term prognosis, with the 5-year survival remaining unsatisfactory^[12,13]^. Therefore, novel therapeutic strategies targeting molecular vulnerabilities are urgently needed.

Oncolytic viruses (OVs) represent a promising class of anti-cancer agents that selectively replicate in and lyse tumor cells while sparing normal tissue^[14]^. In addition to direct oncolysis, OVs enhance systemic antitumor immunity by promoting antigen release and immune activation. Several OVs - including adenovirus, herpes simplex virus, and vaccinia virus - have entered clinical trials^[15-17]^. Bladder cancer is particularly amenable to OV therapy due to the ease of local administration and its immunogenic nature^[18]^. Combinatorial strategies involving OVs and immune checkpoint inhibitors or chemotherapy have shown synergistic potential^[19-21]^. However, a detailed understanding of host-virus interactions is essential to optimize efficacy.

The endoplasmic reticulum (ER) plays a central role in ensuring proper protein folding and post-translational processing. When ER homeostasis is disturbed, unfolded or misfolded proteins accumulate, leading to ER stress (ERS)^[22]^. This condition activates several adaptive pathways, including the unfolded protein response (UPR), ER overload response (EOR), and the sterol regulatory element-mediated cascade, among which UPR functions as the primary regulatory mechanism^[23,24]^. UPR is a highly conserved signaling network in eukaryotic cells that monitors protein folding and synthesis within the ER to restore homeostasis. It is mediated by three key transmembrane proteins: Protein kinase RNA-like ER kinase (PERK), Inositol-requiring enzyme 1 alpha (IRE1α), and Activating transcription factor 6 alpha (ATF6α)^[25]^. Additionally, the ER-resident molecular chaperone immunoglobulin heavy chain-binding protein (Bip), also known as HSPA5 or GRP78, plays a pivotal role in both protein folding and UPR regulation^[26]^. Under stress, Bip dissociates from these proteins, activating them. PERK reduces protein synthesis via eIF2α phosphorylation and promotes ATF4 expression. ATF6 and IRE1α enhance expression of UPR-related genes like *XBP1* and *CHOP*, improving protein folding capacity^[27,28]^. Viral infections can exploit the UPR to facilitate replication^[29]^. Oncolytic virus M1 induces ERS in tumor cells, leading to apoptosis, suggesting that modulating UPR can enhance its therapeutic efficacy^[30,31]^.

M1 is a naturally occurring alphavirus that selectively replicates in tumor cells and spares normal tissues^[32]^. Our previous study demonstrated that M1 preferentially kills MIBC cells with low zinc finger antiviral protein (ZAP) expression. ZAP knockdown restores M1 sensitivity, while overexpression confers resistance. In murine models, systemic M1 administration significantly reduced tumor growth and prolonged survival, outperforming cisplatin. Clinically, ZAP was low in 45.6% of MIBC cases, especially in advanced tumors^[33]^. However, the mechanism of M1’s selective cytotoxicity remains incompletely understood. In this study, we identify the IRE1α branch of the UPR as a key modulator of M1 sensitivity in MIBC. Transcriptomic, *in vitro*, and *in vivo* data show that impaired IRE1α signaling enhances M1-induced ER stress and apoptosis. Combination treatment with the IRE1α inhibitor STF significantly improved antitumor effects in mice without additional toxicity. Clinically, IRE1α was downregulated in primary bladder cancer cells and associated with worse disease-free survival in The Cancer Genome Atlas (TCGA) data. These findings suggest that targeting IRE1α can augment M1-based virotherapy in bladder cancer.

## METHODS

### Cell culture

Bladder cancer cell lines including SV-HUC-1, TCC, EJ, RT-4, 5637, SCaBER, UM-UC-3, T24, and BIU87 were obtained from ATCC (Manassas, VA, USA), the Shanghai Cell Bank (Shanghai, China), and the Guangzhou Institutes of Biomedicine and Health (Guangzhou, China). Human primary normal bladder epithelial cells (HBSMCs) were purchased from ScienCell (Carlsbad, CA, USA), while human primary bladder tumor cells were isolated from surgical specimens obtained from consented patients undergoing radical cystectomy at the Third Affiliated Hospital of Sun Yat-sen University. The human study was approved by the Institutional Review Board of the same hospital. All established cell lines were cultured according to the suppliers’ instructions in DMEM complete medium supplemented with 10% fetal bovine serum (FBS), 100 U/mL penicillin, and 100 µg/mL streptomycin. The human primary bladder epithelial cell line HBSMC was cultured in F-12 medium with the same supplements. Cells were maintained in a humidified incubator at 37 °C with 5% CO_2_. Cells were observed under an inverted microscope and passaged when they reached ~80% confluence; only cells in the logarithmic growth phase were used for subsequent experiments.

### Primary cell isolation

For the isolation and culture of human primary bladder cancer cells, culture dishes were pre-coated with type I collagen (5 µg/cm^2^) at room temperature for 1 h, rinsed once with sterile PBS, and air-dried in a biosafety cabinet. Tissue samples (1-2 cm^3^) were minced into small fragments. Fragments were digested with 5 mL trypsin at 37 °C for 30 min, with gentle mixing every 5 min. In the final minute, DNase I was added to a final concentration of 80 µg/mL. Digestion was terminated with 1 mL FBS, followed by centrifugation at 1,500 rpm for 5 min at 4 °C. The cell pellet was resuspended in 4 mL serum-free DMEM containing 80 µg/mL DNase I and 3 mM MgSO_4_, then gently pipetted to disperse single cells. The suspension was filtered through a 200-mesh sieve to remove undigested tissue and centrifuged again at 1,000 rpm for 5 min at 4 °C. The resulting cell pellet was resuspended in 10% FBS F12/DMEM medium and seeded onto collagen-coated dishes. Cells were cultured at 37 °C with 5% CO_2_ and 95% humidity. After 24 h, non-adherent cells and debris were removed by PBS washing, and fresh medium was added. Medium was changed every 2-3 days, and drug treatments were administered once cells reached ~60% confluence.

### siRNA transfection

IRE1α expression was silenced using siIRE1α (RiboBio, Guangzhou, China) transfection with Lipofectamine RNAiMAX (13778030, Invitrogen, Waltham, MA, USA), following the manufacturer’s instructions. Cells were seeded into 6-well plates in antibiotic-free DMEM containing 10% FBS and allowed to adhere overnight, reaching 40%-60% confluency at the time of transfection. For each well, 2 µL of Lipofectamine RNAiMAX was diluted in 200 µL of Opti-MEM medium and gently mixed. Separately, IRE1α siRNA was diluted in 200 µL Opti-MEM to a final concentration of 50 nM. The diluted Lipofectamine and siRNA solutions were then combined (1:1 ratio) and incubated at room temperature for 15 min. The 400 µL transfection mixture was added and incubated for 48 h. After transfection, the medium was replaced with complete DMEM, and cells were used for subsequent experiments.

The sequences of the siRNAs targeting IRE1α were as follows: SiIRE1α-001 CCATGGATCATGTCTCCTT SiIRE1α-002 GCTATGACCTCGGTCCTAA SiIRE1α-003 GCTACAGGCGTTACAAATA

A non-targeting scrambled siRNA (siNC) was used as a negative control, and all siRNAs were synthesized by RiboBio (Guangzhou, China).

### Animal models

All animal experiments were approved by the Animal Ethics Committee of Lai’an Technology (Guangzhou) Co., Ltd. Female BALB/c-nu/nu nude mice (4-6 weeks old) were purchased from the Guangdong Animal Center and maintained under specific pathogen-free (SPF) conditions. Human bladder cancer UM-UC-3 cells in the logarithmic growth phase were harvested using trypsin, neutralized with complete DMEM medium, and centrifuged at 600 × *g*. The cell pellet was resuspended in phosphate-buffered saline (PBS), and the concentration was adjusted to 1-3 × 10^7^ cells/mL.

Prior to surgery, mice were anesthetized with an intraperitoneal injection of 4.3% chloral hydrate at a dose of 0.1 mL per 10 g of body weight. Once anesthesia was confirmed by the loss of limb reflexes, mice were fixed in a supine position on a sterile surgical platform. A lower midline abdominal incision was made after disinfection with iodophor, and the bladder was gently exteriorized. After expressing the urine, approximately 50 µL of the prepared UM-UC-3 cell suspension was slowly injected into the bladder wall using a 1 mL syringe with a 16-gauge needle. Care was taken to avoid injecting into the bladder lumen or penetrating the entire bladder wall; successful injection was indicated by a localized bulge in the bladder wall without leakage from the urethra. The bladder was repositioned into the abdominal cavity, and the peritoneal and skin layers were closed with 5-0 absorbable sutures. Mice were kept warm during recovery and returned to their cages once fully awake. One week post-injection, mice were randomly selected for necropsy to verify the successful establishment of orthotopic bladder tumors.

### Oncolytic M1 virus

To amplify the M1 virus, Vero cells were cultured in OPTIPRO-SFM medium supplemented with 4 mM GlutaMAX. When cell confluence reached approximately 80%, M1 virus was added at a multiplicity of infection (MOI) of 0.01. After about 36 h, when most cells showed cytopathic effects (CPEs) but had not yet detached, the culture supernatant was collected. The supernatant was centrifuged at 1,000 × *g* for 5 min at 4 °C to remove cell debris, then aliquoted and stored at -80 °C for future use.

### Virus titers

The viral titer of the M1 virus was determined using the TCID_50_ method. BHK-21 cells were seeded into 96-well plates at a density of 2 × 10^5^ cells/mL. M1 virus was diluted tenfold in serum-free DMEM from 10^-1^ to 10^-8^. CPE was observed daily for 3 days, and the number of wells showing CPE at each dilution was recorded. Viral titer was calculated using the Karber method with the formula: log TCID_50_ = L - D × (S - 0.5), where L is the log of the highest dilution (-1), D is the log dilution interval (-1), and S is the cumulative proportion of positive wells. Based on the calculation (log TCID_50_ = -3.625), the viral titer was determined to be 10^-3.625^ TCID_50_ per 0.1 mL, equivalent to 3.9 × 10^5^ TCID_50_/mL.

### MTT assay

Cells were seeded in 24-well plates at a density of 4 × 10^4^ cells/mL. The oncolytic virus M1 was added at MOIs of 0.001, 0.01, 0.1, 1, and 10, with three replicate wells per condition, along with a control group of untreated cells. After 48 h of infection, 100 µL of MTT solution (02102227-CF, MP Biomedicals, Illkirch-Graffenstaden, France) was added to each well, and plates were incubated at 37 °C for 4 h. The supernatant was then removed, and 500 µL of DMSO was added to each well, followed by 5 min of shaking on a plate shaker. Absorbance at 570 nm was measured using a microplate reader. Each experiment was performed in triplicate, and relative cell viability was calculated as: (OD of treated group/OD of control group) × 100%.

### Immunofluorescence

Cells were seeded into 6-well plates at a density of 5 × 10^4^ cells/mL. After incubation with M1 virus at a MOI of 1 for 48 h, cells were stained with Hoechst 33342 at a final concentration of 1 µg/mL for 20 min. Nuclear morphology was then observed and imaged under a fluorescence microscope.

### Immunohistochemistry

Paraffin-embedded tissue sections (5 µm thick) were incubated at 60 °C for 30 min and deparaffinized with xylene and rehydrated through graded ethanol. Endogenous peroxidase activity was blocked with 3% methanol-H_2_O_2_ for 20 min. Antigen retrieval was performed by boiling the sections in 0.01 M citrate buffer (pH 6.0) using a cycle of heating for 2 min, simmering for 6 min, and repeating once, then allowing the slides to cool naturally to room temperature. Sections were blocked with normal non-immune serum at room temperature for 20 min. Slides were incubated with primary antibodies Ki-67 (1:400, #9449, Cell Signaling Technology, Irving, TX, USA) and Cleaved-Caspase-3 (1:400, #9664, Cell Signaling Technology, Irving, TX, USA) overnight at 4 °C. After rinsing PBS, slides were incubated with HRP-conjugated secondary antibodies at 37 °C for 30 min. Immunoreactivity was visualized using DAB substrate. Slides were counterstained with hematoxylin, dehydrated in ascending ethanol concentrations, cleared in xylene, and coverslipped using a permanent mounting medium. Immunostaining images were captured using an Olympus microscope. Quantitative analysis of brown staining was performed using Image-Pro Plus 6.0 (IPP 6.0), and average optical density was calculated to assess protein expression.

### Western blot

Cells were lysed with lysis buffer on ice for 5 min. Cells were scraped and centrifuged at 12,000 × *g* for 10 min at 4 °C. The supernatant containing total protein was collected to measure protein concentrations using the BCA assay according to the manufacturer’s instructions. Equal amounts of protein (40-80 µg) were loaded and electrophoresed at a constant voltage of 120 V. After electrophoresis, proteins were transferred onto PVDF membranes. Membranes were blocked with 5% non-fat milk in TBST for 2 h at room temperature, then incubated overnight at 4 °C with primary antibodies diluted in 5% BSA in TBST. Antibodies were listed as following: IRE1α (1:1,000, ab96481, Abcam, Cambridge, UK), PERK (1:1,000, #5683, Cell Signaling Technology, Irving, TX, USA), ATF6α (1:1,000, #65880, Cell Signaling Technology, Irving, TX, USA), eIF-2α (1:1,000, #5324, Cell Signaling Technology, Irving, TX, USA), phosphorylated eIF-2α (1:1,000, #3398, Cell Signaling Technology, Irving, TX, USA), GAPDH (1:1,000, AP0060, Bioworld), β-actin (1:1,000, AP0063, Bioworld), JNK (1:1,000, #9252, Cell Signaling Technology, Irving, TX, USA), phosphorylated JNK (1:1,000, #925, Cell Signaling Technology, Irving, TX, USA), M1 E1 and NS3 (1:1,000, produced by Beijing Protein Innovation), CHOP (1:1,000, ab11419, Abcam, Cambridge, UK), XBP1 (1:1,000, #40435, Cell Signaling Technology, Irving, TX, USA), ZAP (1:1,000, #PA5-31650, Thermo Scientific, USA), Caspase-12 (1:1,000, #58208, Cell Signaling Technology, Irving, TX, USA) and cleaved-Caspase-3 (1:1,000, #9664, Cell Signaling Technology, Irving, TX, USA). The next day, membranes were washed three times with TBST and incubated with HRP-conjugated secondary antibodies diluted 1:1,000 in blocking buffer for 1 h at room temperature. Membranes were incubated with enhanced chemiluminescence (ECL) solution, and bands were visualized using the Bio-Rad ChemiDoc XRS+ imaging system. Band intensities were quantified using Image Lab 4.0 software.

### Real-time qRT-PCR

Total RNA was extracted using TRIzol reagent. Cells were lysed in 1 mL TRIzol (11TRIMPR01, MP Biomedicals, Illkirch-Graffenstaden, France) on ice, and incubated at room temperature for 5 min. Chloroform (200 µL) was added. After vigorous shaking for 30 s and a 5-minute rest at room temperature, samples were centrifuged at 12,000 × *g* for 15 min at 4 °C. The upper aqueous phase was collected, and RNA was precipitated with 500 µL isopropanol, incubated at room temperature for 10 min, and centrifuged at 12,000 × *g* for 10 min at 4 °C. The RNA pellet was washed with 75% ethanol, air-dried, and dissolved in 20-80 µL of DEPC-treated water. For reverse transcription, 1 µg of total RNA was denatured at 65 °C for 5 min, and then reverse-transcribed using RevertAid Reverse Transcriptase (EP0441, Thermo Fisher Scientific, Waltham, MA, USA). Real-time PCR was performed on an ABI 7500 Fast system using the following cycling conditions: 95 °C for 15 min, followed by 40 cycles of 95 °C for 10 s and 61 °C for 30 s. Primers used were listed as follows: IRE1α: Forward CGCTTCGGAAACCTGGAAGA; Reverse TCCTCGTCGTTCTTCCAGTAGC. PERK: Forward GAGCACTGGAGATTCGGTGAA; Reverse CGGCAGGTCTTCTTCTTCTG. ATF6: Forward GACCCGGAGGAGCTGGAGTA; Reverse GTTGATGGCTGGCTGTCTTTG. Melt curve analysis was performed to ensure specificity. Relative gene expression was calculated using the 2^-ΔΔCt^ method, normalized to a housekeeping gene.

### Transmission electron microscopy

Cells were seeded into 6-well plates at a density of 5 × 10^4^ cells/mL. After 24 h of adherence, cells were infected with the M1 virus at an MOI of 1. At 12 and 24 h post-infection, cells were harvested by scraping, centrifuged at 3,000 rpm for 5 min, and the supernatant was discarded. Cells were fixed sequentially with 2.5% glutaraldehyde and 1% osmium tetroxide, followed by graded dehydration using ethanol and acetone. Samples were embedded in Epon 812 resin, stained with uranyl acetate and lead citrate, and examined under a transmission electron microscope to observe ultrastructural changes.

### Statistical analysis

Quantitative data following a normal distribution were presented as mean ± standard deviation (SD). Statistical analyses were conducted using SPSS 13.0. Group comparisons were performed using the *t*-test or one-way ANOVA with Dunnett’s post hoc test. Correlations were assessed using Pearson’s correlation coefficient. All experiments were independently repeated at least three times. Normality was confirmed prior to analysis, and *P* < 0.05 was considered statistically significant.

## RESULTS

### M1 virus induces selective cytotoxicity in MIBC cells via ER stress

**Figure 1:**
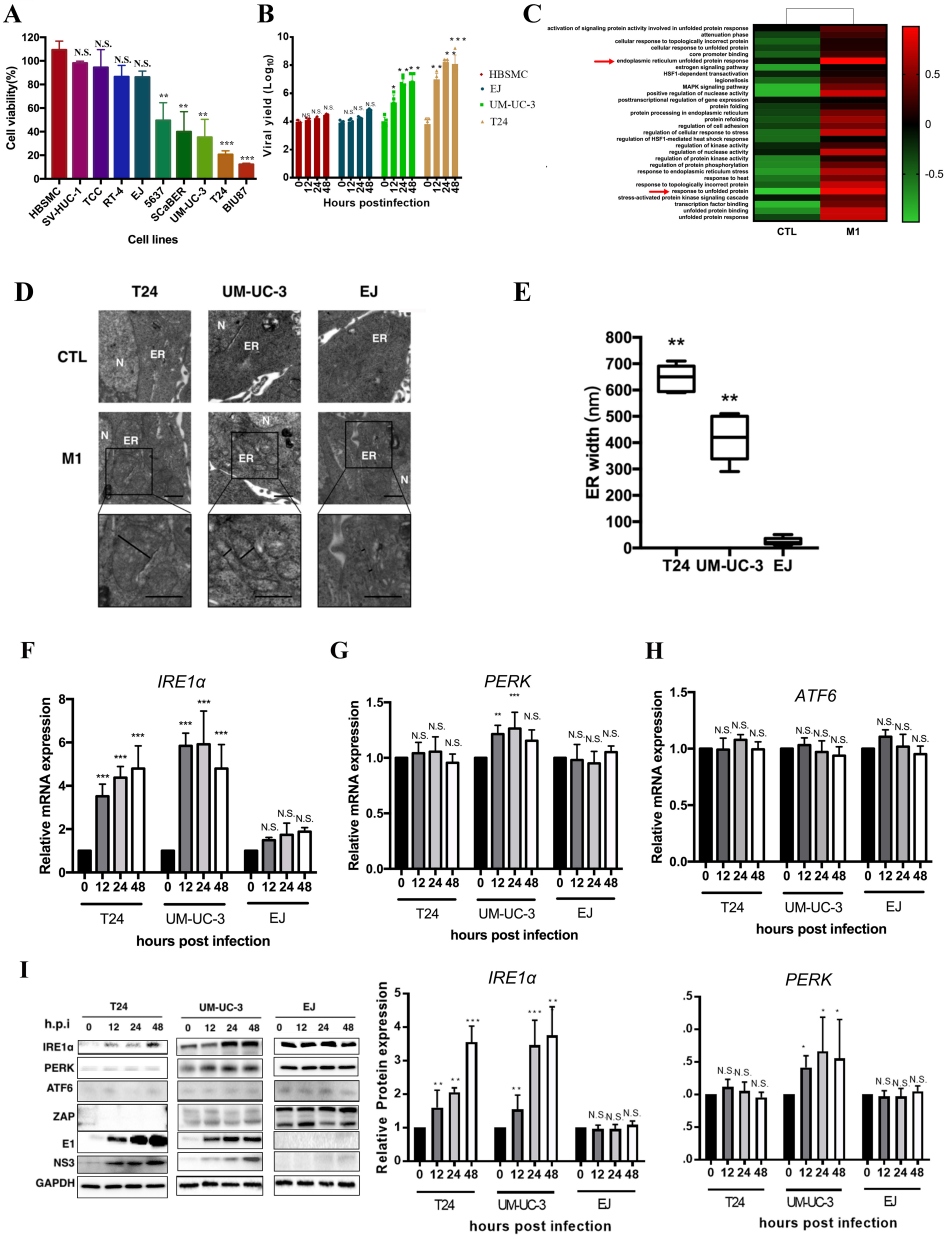
M1 virus induces ER stress selectively in MIBC cells and activates the IRE1α pathway. (A) Cell viability of bladder cancer cell lines and normal urothelial cells after M1 infection (MOI = 10, 48 h); (B) Time course of viral yield in four representative cell lines following M1 virus infection (MOI = 10); (C) Pathway enrichment heatmap based on RNA-seq data comparing M1-treated *vs.* control tumor cells; ER stress and UPR-related pathways are upregulated (red arrows); (D) TEM of ER morphology in T24, UM-UC-3, and EJ cells treated with control or M1. ER: endoplasmic reticulum; N: nucleus; (E) Quantification of ER width based on TEM images; (F-H) Relative mRNA expression of UPR pathway genes *IRE1α*, *PERK*, and *ATF6* at 0, 12, 24, and 48 h post-infection in T24, UM-UC-3, and EJ cells; (I) Immunoblot analysis of ER stress proteins (IRE1α, PERK, ATF6), antiviral protein ZAP, and M1 viral proteins (E1, NS3) in the same cells over 48 h post-infection. GAPDH was used as a loading control. The expression levels of IRE1α and PERK in T24, UM-UC-3, and EJ cells were further quantified by densitometric analysis. ^*^P < 0.05, ^**^P < 0.01, ^***^P < 0.001; N.S.: not significant. MIBC: Muscle-invasive bladder cancer; IRE1α: inositol-requiring enzyme 1 alpha; MOI: multiplicity of infection; TEM: transmission electron microscopy; UPR: unfolded protein response; ZAP: zinc finger antiviral protein; PERK: protein kinase RNA-like ER kinase.

To investigate the mechanism underlying the selective oncolytic effect of M1 virus in MIBC, we first evaluated its cytotoxicity. M1 induced significantly higher cell death in medium- (UM-UC-3, SCaBER, 5637) to high-sensitive cell lines (T24, BIU87) compared to low-sensitive lines (EJ, RT4, TCC) and controls (HBSMC, SV-HUC-1) [Figure 1A]. Furthermore, we tracked viral titers over time to assess replication dynamics. A progressive increase in viral production was observed in the sensitive T24 and UM-UC-3 cell lines, indicating active and efficient viral replication. Conversely, in the less responsive EJ and TCC cells, viral levels remained largely stable throughout the observation period, suggesting a limited capacity to support M1 propagation [Figure 1B]. To explore the mechanism of this selective vulnerability, pathway enrichment analysis based on published RNA-seq data from M1-infected UM-UC-3 cells^[30]^ showed significant upregulation of gene sets related to ER stress and UPR [Figure 1C]. TEM confirmed ER swelling in M1-treated T24 and UM-UC-3 cells, but not in EJ cells [Figure 1D]. Quantified ER width was significantly increased in T24 and UM-UC-3, but unchanged in EJ [Figure 1E]. qRT-PCR showed IRE1α was strongly upregulated in T24 and UM-UC-3, but not EJ [Figure 1F]; PERK was modestly increased in UM-UC-3 [Figure 1G], and ATF6 showed no change across all lines [Figure 1H]. Western blot analysis revealed a time-dependent induction of IRE1α in both T24 and UM-UC-3 cells following M1 infection, with no significant changes observed in EJ cells. PERK expression was notably increased in UM-UC-3 cells at 12-24 h post-infection, while remaining relatively stable in T24 and EJ. ATF6 levels remained unchanged across all three cell lines during the infection time course [Figure 1I]. Our previous study reported low ZAP in UM-UC-3^[30]^. UM-UC-3 mainly expressed ZAP-long, but M1 infection induced a shift to ZAP-short. EJ dominantly expressed ZAP-short, with no isoform change. M1 viral proteins (E1, NS3) were abundant in sensitive cells but limited in EJ [Figure 1I]. These results suggest ER stress, particularly via the IRE1α branch of UPR, mediates M1’s selective cytotoxicity in MIBC.

### Induction of ER stress enhances M1 virus cytotoxicity via the IRE1α pathway in MIBC cells

**Figure 2:**
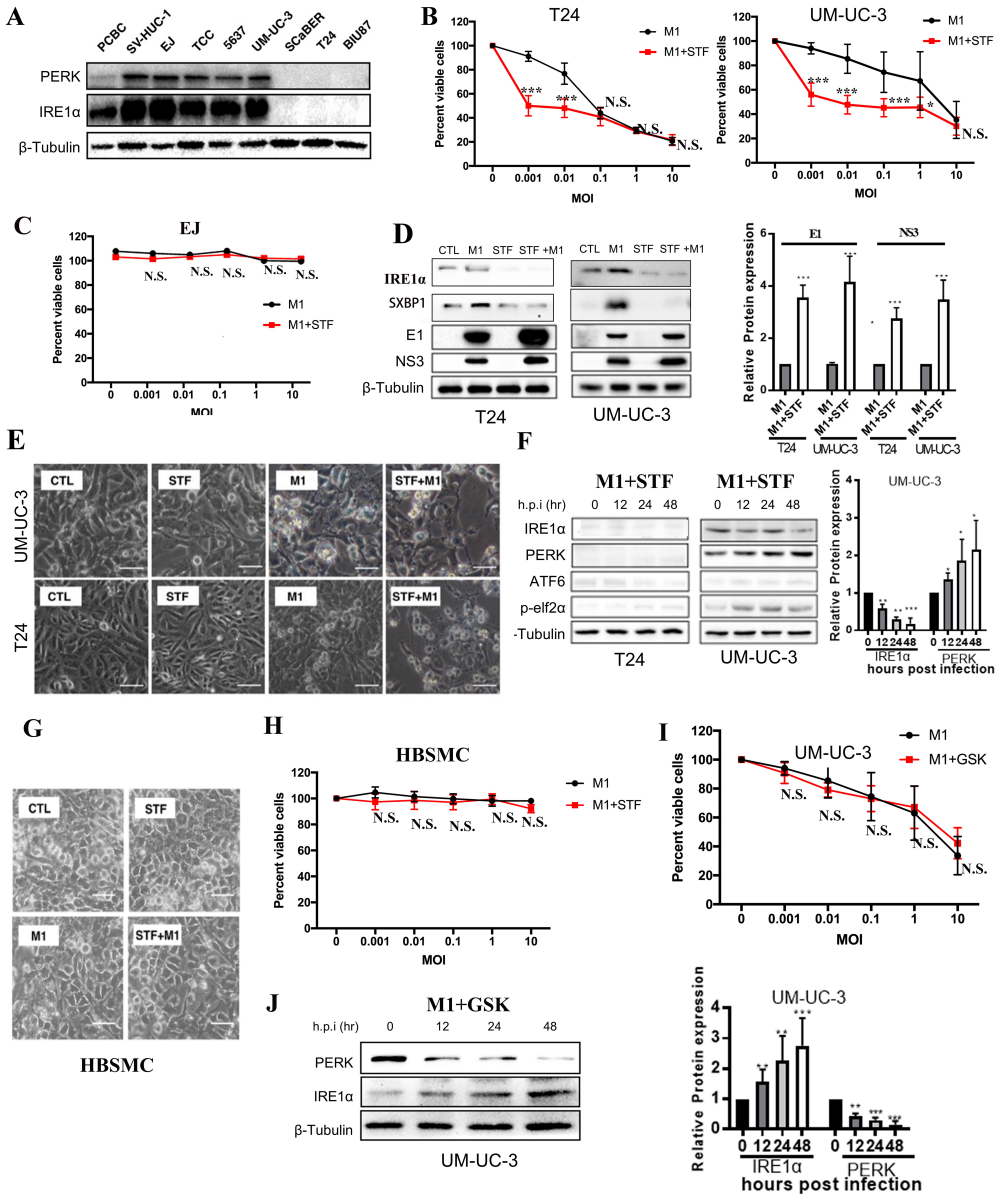
ER stress induction enhances M1 virus-mediated cytotoxicity through the IRE1α pathway in MIBC cells. (A) Western blot of PERK and IRE1α expression across normal and bladder cancer cell lines; (B and C) Cell viability of T24, UM-UC-3, and EJ cells treated with M1 alone or M1 + STF (10 µM) for 48 h; (D) T24 and UM-UC-3 cells were treated with STF (10 µM), M1 (MOI = 0.01), or a combination of both. Protein levels of IRE1α, sXBP1, and M1 viral proteins E1 and NS3 were analyzed by Western blot, followed by quantification of E1 and NS3 expression; (E) Morphological changes in T24 and UM-UC-3 cells treated with control (CTL), STF (10 μM), M1 (MOI = 0.01), or STF + M1 (scale bar = 100 μm); (F) Western blot analysis of ER stress markers (IRE1α, PERK, ATF6, and p-eIF2α) in T24 and UM-UC-3 cells over time following co-treatment with STF (10 μM) and M1 (MOI = 0.01). Densitometric analysis of IRE1α and PERK expression in UM-UC-3 cells is also shown; (G and H) Phase-contrast images and viability of HBSMCs treated with M1 (MOI = 10) and/or STF (scale bar = 100 μm); no significant cytotoxicity observed; (I) Cell viability in UM-UC-3 cells treated with M1 (MOI = 0.01) alone or M1 + GSK (10 μM) shows no enhancement of cytotoxicity; (J) Western blot of PERK and IRE1α over time in UM-UC-3 cells treated as in (I). ^*^P < 0.05, ^**^P < 0.01, ^***^P < 0.001; N.S.: not significant. ER: Endoplasmic reticulum; IRE1α: inositol-requiring enzyme 1 alpha; MIBC: muscle-invasive bladder cancer; PERK: protein kinase RNA-like ER kinase; MOI: multiplicity of infection; sXBP1: spliced XBP1; HBSMCs: human primary normal bladder epithelial cells.

We next examined whether enhancing ER stress could augment the cytotoxicity of M1 virus in bladder cancer cells. Western blot showed that highly M1-sensitive MIBC cells (T24, BIU87) and the moderately sensitive SCaBER cells had minimal IRE1α and PERK expression, whereas non-tumorigenic cells and low/moderately sensitive lines (EJ, TCC, UM-UC-3, 5637) displayed elevated levels [Figure 2A].

To test if IRE1α inhibition enhances M1-induced cytotoxicity, T24 cells (lacking IRE1α expression) and UM-UC-3 cells (expressing IRE1α) were pretreated with STF083010 (STF), a selective inhibitor of the RNase activity of IRE1α, before M1 infection. STF significantly enhanced M1-induced cell death at low MOIs in both lines, but not at MOI 1 or 10 [Figure 2B]. In contrast, EJ cells were resistant to M1-induced cytotoxicity with or without STF treatment, showing no significant reduction in cell viability across all MOIs [Figure 2C]. STF (10 μM) suppressed IRE1α signaling in T24 and UM-UC-3 cells, as shown by reduced IRE1α and sXBP1 levels. Co-treatment with M1 virus significantly increased E1 and NS3 protein expression compared to M1 alone, suggesting that IRE1α inhibition may enhance viral protein accumulation [Figure 2D]. Morphologically, the STF + M1 group showed more evident cell damage and death, suggesting that IRE1α pathway inhibition enhances the oncolytic activity of M1 virus [Figure 2E]. Following M1 and STF treatment, IRE1α expression progressively decreased in UM-UC-3 cells, while remaining low in T24 cells and nearly undetectable at 48 h. Quantitative analysis confirmed sustained IRE1α downregulation and gradual PERK upregulation in UM-UC-3 cells, with ATF6 levels unchanged [Figure 2F].

Importantly, M1 alone or with STF did not affect SV-HUC-1 cell morphology or viability [Figure 2G and H], supporting tumor selectivity. To further probe the UPR, we inhibited the PERK pathway using GSK2606414. In UM-UC-3, GSK2606414 co-treatment did not alter M1 cytotoxicity [Figure 2I]. Western blot and densitometric analysis demonstrated that in UM-UC-3 cells co-treated with the PERK inhibitor GSK2606414 and M1 virus, PERK expression declined over time, whereas IRE1α levels increased [Figure 2J]. These findings suggest that blocking the PERK pathway does not potentiate M1-induced oncolysis, suggesting that M1’s oncolytic efficacy is not dependent on the PERK pathway.

### IRE1α knockdown enhances M1-induced apoptosis and viral protein expression

**Figure 3:**
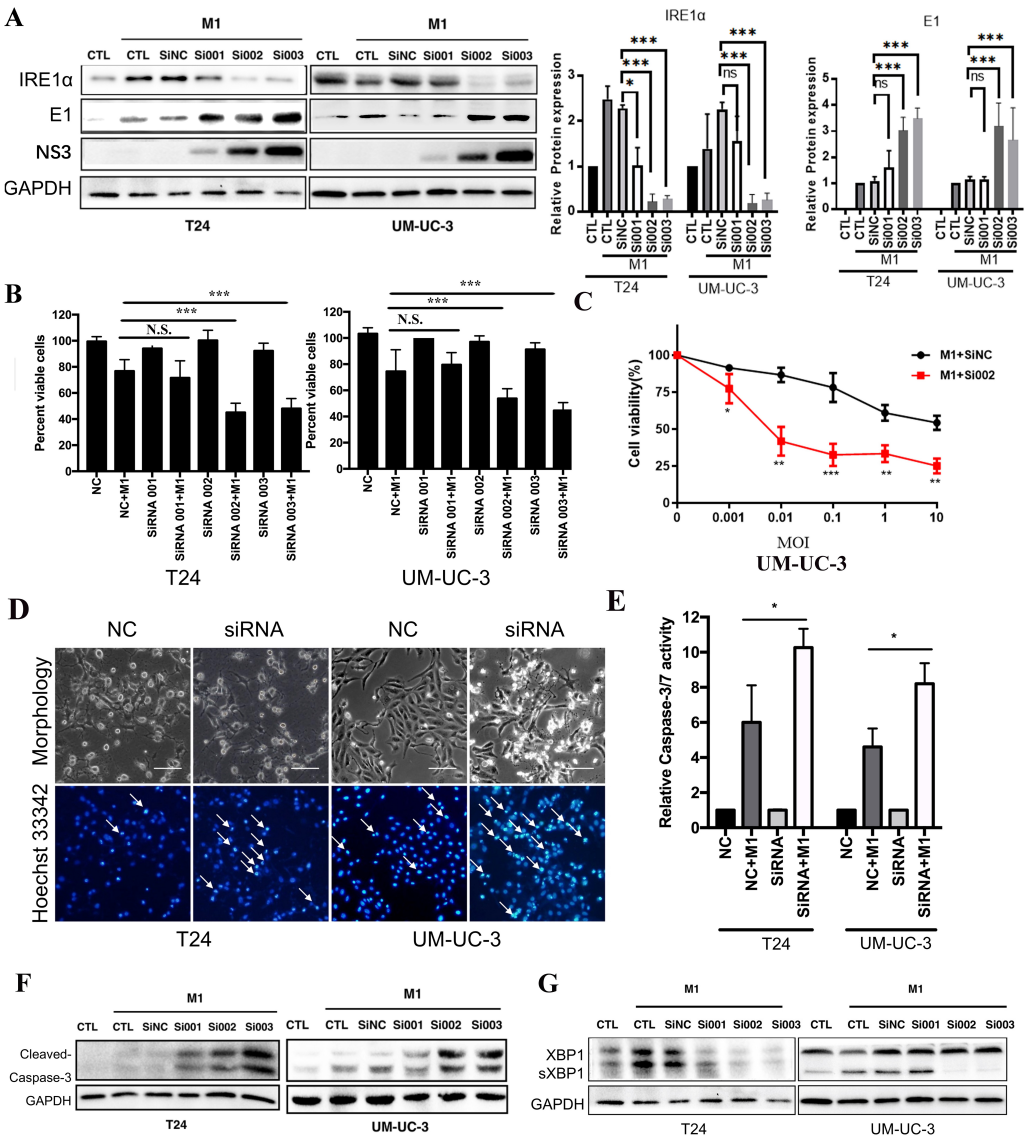
IRE1α silencing enhances M1-mediated apoptosis and viral replication in bladder cancer cells. Western blot analysis and densitometric quantification of IRE1α, E1, and NS3 protein levels in T24 and UM-UC-3 cells transfected with control siRNA (siNC) or IRE1α-specific siRNAs (si001, si002, si003), followed by M1 virus infection. GAPDH was used as a loading control; (B) Quantification of cell viability after M1 treatment in the presence or absence of IRE1α knockdown; (C) Cell viability of UM-UC-3 cells after IRE1α knockdown (si002) or control (siNC) combined with M1 treatment for 48 h; (D) Morphology and Hoechst 33342 nuclear staining of T24 and UM-UC-3 cells showing apoptotic features (arrows) enhanced by IRE1α knockdown (siRNA002); (E) Relative caspase-3/7 activity measurements confirm enhanced apoptotic signaling with IRE1α knockdown (siRNA002); (F) Western blot detection of cleaved caspase-3 in T24 and UM-UC-3 cells, showing increased apoptosis in siRNA + M1 groups; (G) Immunoblot showing elevated M1 viral proteins (E1 and NS3) in IRE1α knockdown groups, indicating increased viral replication. ^*^P < 0.05, ^**^P < 0.01, ^***^P < 0.001; N.S.: not significant. IRE1α: Inositol-requiring enzyme 1 alpha.

T24 cells, highly sensitive to M1, initially lacked detectable IRE1α expression; however, M1 infection notably induced IRE1α, suggesting its involvement in the cellular response. To explore its role in viral regulation, we transfected T24 and UM-UC-3 cells with three independent siRNAs targeting IRE1α (si001, si002, si003). Western blot and densitometric analyses confirmed efficient knockdown of IRE1α in both cell lines. Notably, IRE1α depletion markedly increased the expression of the M1 viral proteins (E1 and NS3) compared to control cells [Figure 3A]. Correspondingly, cell viability was significantly reduced in knockdown groups after M1 infection, indicating enhanced cytotoxicity [Figure 3B]. To assess whether IRE1α silencing enhances M1-induced cytotoxicity, UM-UC-3 cells transfected with siNC or siRNA002 were treated with increasing doses of M1 virus (MOIs). Cell viability declined in both groups in a dose-dependent manner, with si002 cells showing significantly greater susceptibility to M1-induced cell death [Figure 3C]. Hoechst 33342 staining showed increased nuclear condensation and fragmentation in the siIRE1α + M1 groups, consistent with apoptosis [Figure 3D, arrows]. Cleaved caspase-3 levels and caspase-3/7 activity were substantially elevated in IRE1α-silenced cells upon M1 treatment [Figure 3E and F]. In control and SiNC-transfected cells, M1 infection induced robust sXBP1 expression, indicating activation of IRE1α-mediated RNA splicing [Figure 3G]. In contrast, IRE1α knockdown significantly reduced sXBP1 levels in both cell lines, confirming effective suppression of IRE1α endoribonuclease activity. These results demonstrate that IRE1α silencing blocks activation of the IRE1α-XBP1 axis and enhances M1 replication and cytotoxicity in bladder cancer cells.

### IRE1α knockdown amplifies M1-induced ER stress and triggers the CHOP/JNK/Caspase-12 apoptotic pathway

**Figure 4:**
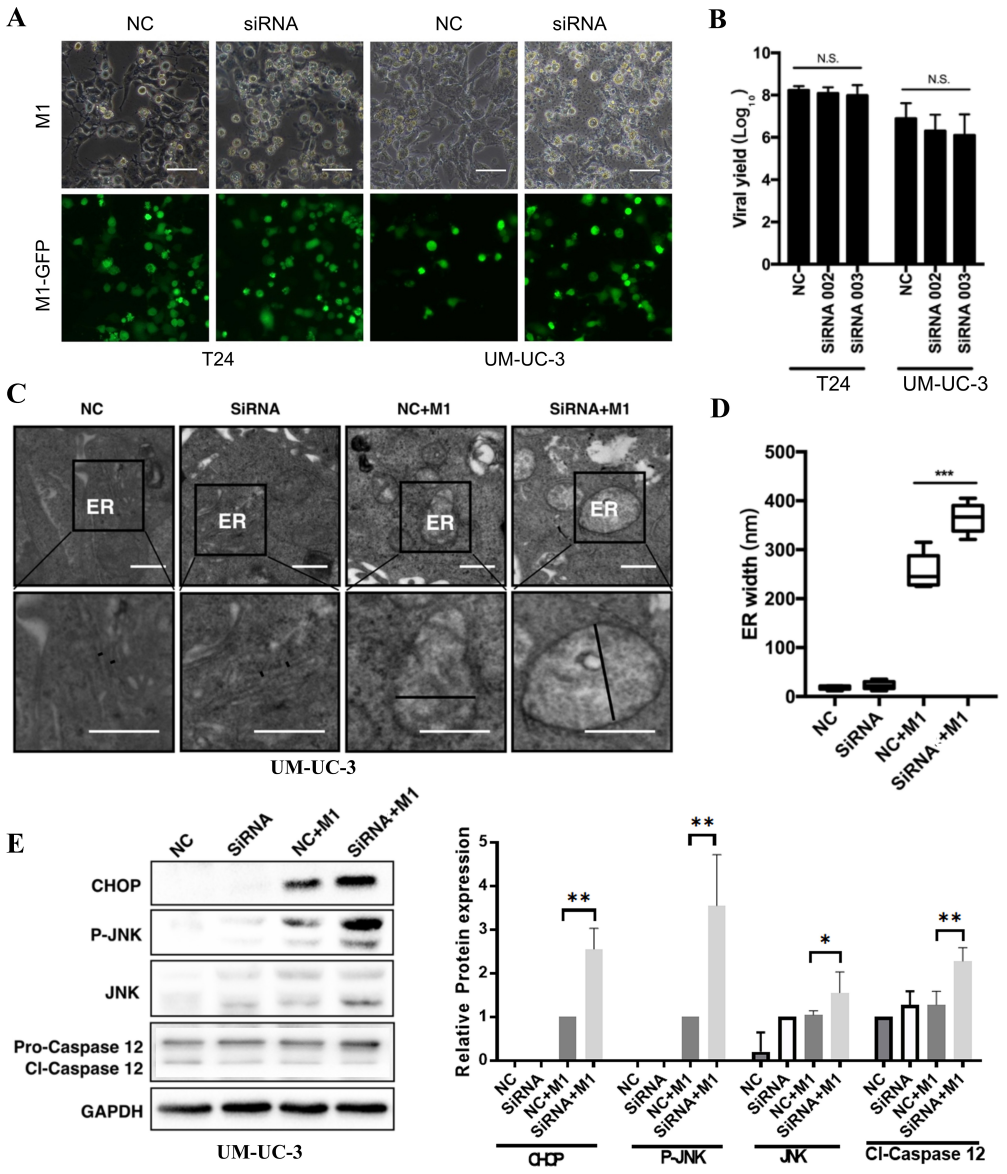
IRE1α knockdown amplifies M1-induced ER stress and activates the CHOP/JNK/caspase-12 pathway. (A and B) Fluorescence imaging and viral yield assay in T24 and UM-UC-3 cells infected with GFP-tagged M1 virus after IRE1α knockdown (siRNA002) or control (NC). Scale bars = 100 μm; (C) TEM of ER morphology in UM-UC-3 cells under the indicated conditions. Marked ER swelling was observed in the siRNA002 + M1 group; (D) Quantification of ER width from TEM images; (E) Western blot and densitometric analysis of ER stress/apoptosis markers CHOP, p-JNK, total JNK, and cleaved caspase-12 (cl-Caspase-12) in UM-UC-3 cells. IRE1α was silenced using siRNA002. GAPDH was used as a loading control. ^*^P < 0.05, ^**^P < 0.01, ^***^P < 0.001; N.S.: not significant. IRE1α: Inositol-requiring enzyme 1 alpha; ER: endoplasmic reticulum; TEM: transmission electron microscopy; p-JNK: phospho-JNK.

To determine how IRE1α knockout contributes to M1-induced cytotoxicity in MIBC cells, we first assessed M1 replication and infectivity following IRE1α knockdown (siRNA002 and siRNA003). Fluorescence microscopy images (representative from siRNA002) and viral yield assays showed no significant difference in GFP expression or viral titers between IRE1α-silenced and control cells, indicating that IRE1α depletion does not impair M1 replication [Figure 4A and B].

Transmission electron microscopy (TEM) showed marked ER swelling in M1-infected and IRE1α-silenced + M1-infected UM-UC-3 cells, while control groups displayed normal ER morphology [Figure 4C]. Quantification demonstrated that M1 infection significantly increased ER width compared to control or IRE1α siRNA (siRNA002) alone, and the combination of IRE1α knockdown and STF treatment further enlarged ER dilation [Figure 4D], indicating elevated ER stress. Western blot confirmed that IRE1α knockdown combined with M1 infection strongly upregulated ER stress-associated apoptotic markers, including CHOP, phosphorylated JNK (p-JNK), and cleaved caspase-12, compared to either treatment alone [Figure 4E]. These results suggest that suppression of the IRE1α branch of the UPR exacerbates M1-induced ER stress and promotes activation of the CHOP/JNK/caspase-12 apoptotic pathway.

### IRE1α inhibitor STF enhances the antitumor efficacy of M1 virus in a bladder cancer xenograft model

**Figure 5:**
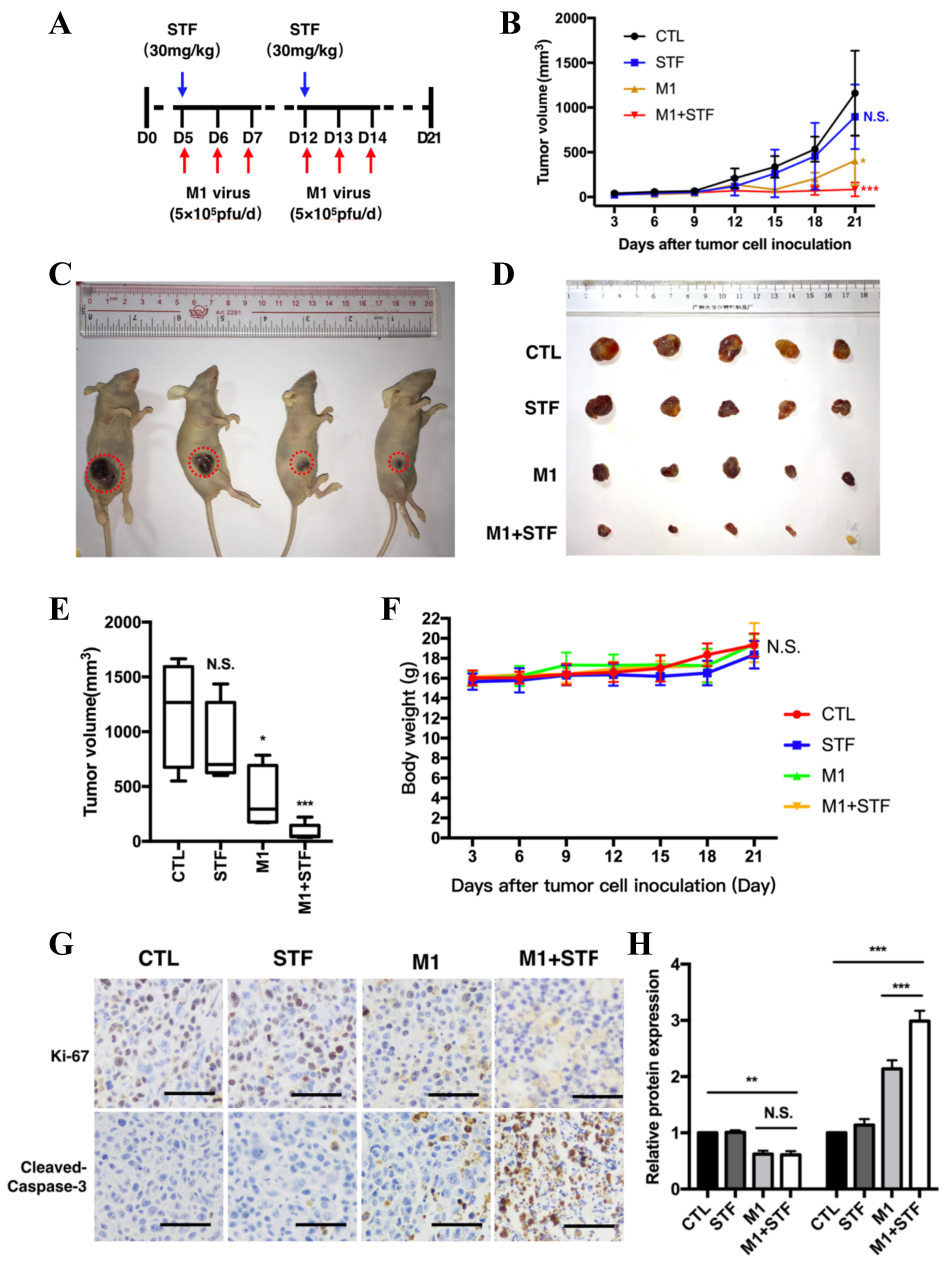
Combination of M1 virus and IRE1α inhibitor STF significantly inhibits bladder tumor growth *in vivo*. Schematic of treatment regimen. STF-083010 (30 mg/kg) was administered intraperitoneally on days 5 and 12. M1 virus (5 × 10^5^ pfu/day) was delivered intravenously for three consecutive days starting on days 6 and 13; (B) Tumor volume growth curves over 21 days; (C and D) Representative images of tumor-bearing mice and harvested tumors on day 21; (E) Final tumor volume measurements; (F) Mouse body weight monitoring across all treatment groups; (G) Immunohistochemical staining of tumor sections for Ki-67 and cleaved caspase-3. Scale bars = 100 μm; (H) Quantification of Ki-67 and cleaved caspase-3–positive cells. ^*^P < 0.05, ^**^P < 0.01, ^***^P < 0.001; N.S.: not significant. IRE1α: Inositol-requiring enzyme 1 alpha.

To verify the synergistic antitumor effect of the IRE1α inhibitor STF on M1 virus therapy *in vivo*, we established a subcutaneous bladder cancer xenograft model in nude mice. Four groups were used: PBS control, STF alone, M1 virus alone, and M1 + STF. Tumor volumes and mouse health were monitored throughout. A lower viral dose (5 × 10^5^ pfu/day) than previous studies (8.7 × 10^7^ pfu/day) was used to highlight potential synergy. STF-083010 was administered intraperitoneally (30 mg/kg) on days 5 and 12, and M1 virus was injected via tail vein on days 6-8 and 13-15 [Figure 5A]. M1 alone significantly suppressed tumor growth compared to PBS and STF alone, while M1 + STF showed the strongest tumor inhibition [Figure 5B]. Tumor images and excised tumors on day 21 confirmed maximum regression in the combination group [Figure 5C and D]. Tumor volume analysis showed a significant reduction in the M1 group (^*^P < 0.05) and a greater reduction in M1 + STF (^***^P < 0.001), while STF alone had no effect [Figure 5E]. Body weight remained stable across groups, indicating good tolerability [Figure 5F]. Immunohistochemistry (IHC) for Ki-67 and cleaved caspase-3 showed decreased proliferation and increased apoptosis in the M1 + STF group compared to others [Figure 5G and H].

### Decreased IRE1α expression in primary bladder cancer and its association with poor prognosis

**Figure 6:**
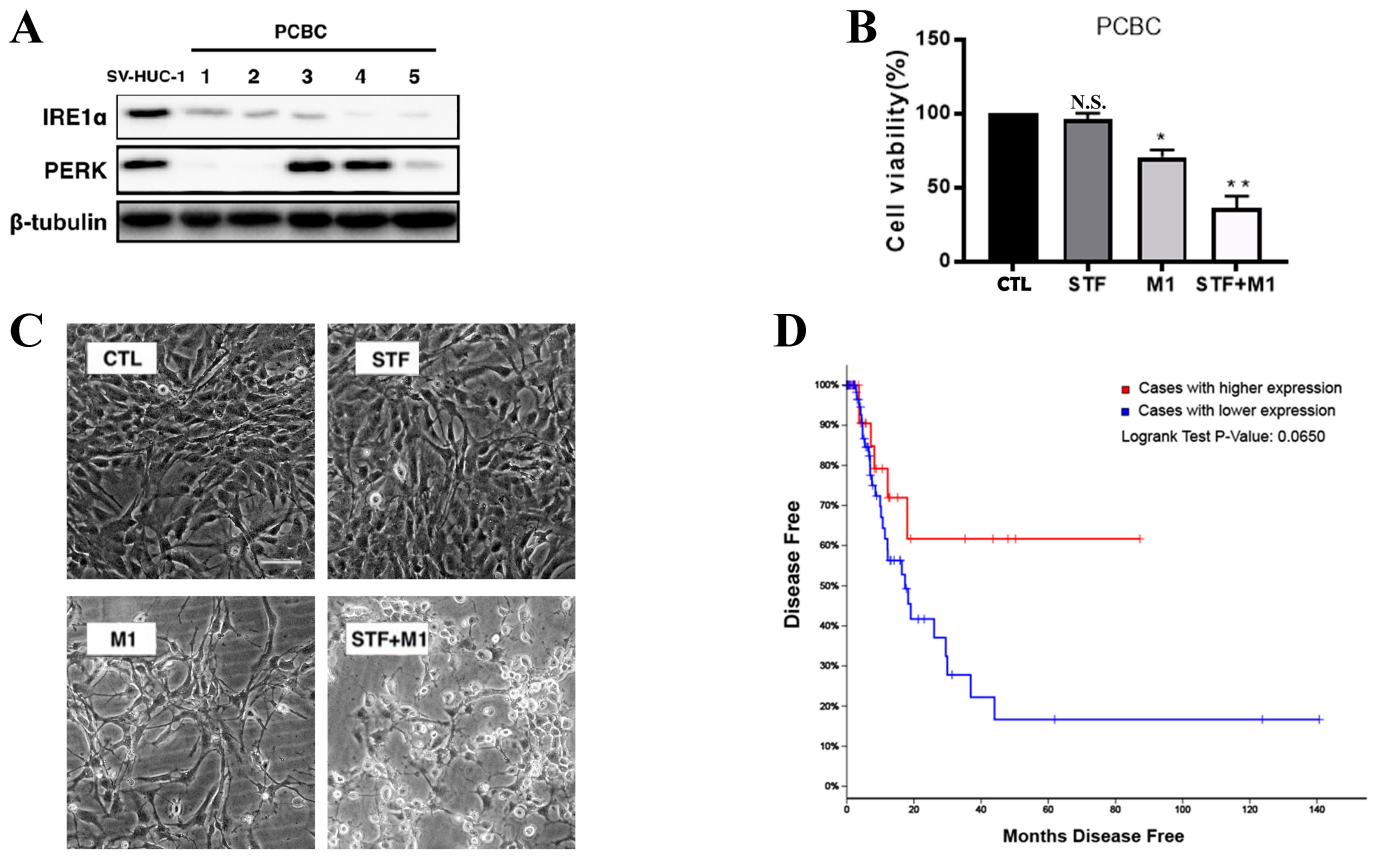
IRE1α is downregulated in bladder cancer and correlates with disease recurrence. (A) Western blot analysis of IRE1α and PERK expression in five PCBC samples compared to the non-tumorigenic bladder epithelial cell line SV-HUC-1. β-tubulin was used as a loading control; (B) Quantification of cell viability in PCBC cells treated with STF (10 μM), M1 (MOI = 0.01), or the combination (STF + M1), relative to the control group (CTL). ^*^P < 0.05, ^**^P < 0.01 *vs.* CTL group; (C) Morphological changes in PCBC cells treated with control (CTL), STF (10 μM), M1 (MOI = 0.01), or STF + M1 (scale bar = 100 μm); (D) Kaplan–Meier disease-free survival analysis of bladder cancer patients from TCGA cohort, stratified by IRE1α expression levels. Patients with high IRE1α expression (red line) exhibited a trend toward poorer prognosis compared to those with low expression (blue line), with a Log-rank *P*-value of 0.0650. IRE1α: Inositol-requiring enzyme 1 alpha; PERK: protein kinase RNA-like ER kinase; PCBC: primary cultured bladder cancer; MOI: multiplicity of infection; TCGA: The Cancer Genome Atlas.

To assess the expression of UPR markers in clinical samples, we analyzed IRE1α and PERK protein levels in five primary cultured bladder cancer (PCBC) cells. IRE1α expression was consistently reduced in all PCBC samples compared to SV-HUC-1 cells [Figure 6A], while PERK levels remained largely unchanged. This suggests a downregulation of the IRE1α branch of the UPR in primary bladder tumors. To investigate the combined effect of IRE1α inhibition and M1 oncolytic virus on primary bladder cancer cells, PCBC cells were treated with STF, M1, or both. M1 treatment alone significantly reduced cell viability compared to the control group (CTL), while the combination treatment (STF + M1) exerted an even greater inhibitory effect [Figure 6B]. Consistently, morphological observations revealed more pronounced cell damage in the STF + M1 group, compared to single-agent treatments [Figure 6C]. Using Analysis of TCGA, Kaplan–Meier analysis of disease-free survival revealed that patients with lower IRE1α expression exhibited a trend toward poorer prognosis compared to those with higher expression (Figure 6D, Log-rank *P* = 0.0650). Together, these data indicate that reduced IRE1α expression may be associated with bladder cancer recurrence and unfavorable outcomes, revealing the potential relevance of targeting this pathway in therapy.

## DISCUSSION

In this study, we uncover a novel mechanism by which M1 virus selectively induces cytotoxicity in MIBC, primarily via disruption of ER homeostasis and activation of the IRE1α-mediated signaling branch of the UPR. OVs like M1 rely on the cellular environment of tumor cells to support replication and lytic activity^[34]^, and our data suggest that differential ER stress responses shape this vulnerability. Using multiple cell lines, we showed M1 infection triggers ER stress in a manner that differentially affects cancer cell lines depending on their intrinsic expression of IRE1α and the isoform balance of the host antiviral factor ZAP. While M1 infection alone was sufficient to trigger ER expansion and pro-apoptotic signaling, combining M1 with IRE1α inhibition further amplified these effects. We also found that STF, an IRE1α pathway inhibitor, markedly potentiated the anti-cancer activity of M1 virus in a bladder cancer xenograft model, without additional toxicity. Furthermore, analysis of primary bladder cancer tissues revealed reduced IRE1α expression relative to normal controls. Consistently, TCGA data indicated a tendency toward shorter disease-free intervals in cases exhibiting low IRE1α levels. These findings raise the possibility that IRE1α downregulation serves as an indicator of adverse clinical outcomes and may help predict responsiveness to M1-based therapeutic strategies.

IRE1α functions as a pivotal sensor in the ER stress response and plays a critical role in regulating the UPR. As a bifunctional protein with both kinase and endoribonuclease activity, IRE1α contributes to ER homeostasis by recognizing the buildup of aberrantly folded proteins within the ER lumen^[35]^. Upon activation, IRE1α triggers a signaling cascade that includes the unconventional splicing of XBP1 mRNA, which produces the active transcription factor XBP1s. This transcription factor promotes the upregulation of molecular chaperones and protein-folding enzymes, thereby mitigating ER stress^[36,37]^. Beyond its canonical role in ER homeostasis, the IRE1α–XBP1 signaling axis also contributes to tumor immune evasion by promoting ER stress–induced dysfunction in dendritic cells and immunosuppressive macrophage polarization^[38-40]^. Highly sensitive MIBC cells, such as T24 and BIU87, exhibit minimal or undetectable IRE1α expression. As a result, they lack the protective UPR response to counteract M1-induced ER stress, leading to pronounced viral replication, ER expansion, and cell death. In contrast, moderately sensitive UM-UC-3 cells express basal IRE1α, which constrains viral protein accumulation and attenuates the full cytotoxic potential of M1. However, these cells remain partially susceptible, potentially due to M1’s ability to shift ZAP isoform expression from ZAP-L to ZAP-S. This shift weakens the antiviral restriction normally conferred by ZAP-L, allowing viral replication to proceed in the ER and triggering ER stress. This isoform regulation may explain why M1 remains effective in UM-UC-3 cells despite their IRE1α expression. EJ cells, which are resistant to M1, express both IRE1α and ZAP-S at baseline, but M1 infection does not induce isoform switching. As a result, these cells remain non-permissive to viral replication, show no expression of viral proteins (E1, NS3), and exhibit minimal ER stress or cytotoxicity. ZAP-L is generally more antiviral, while ZAP-S also has antiviral activity^[41]^. Recent studies suggest it can regulate programmed ribosomal frameshifting (PRF), and its overexpression may disrupt viral RNA structure and suppress replication in SARS-CoV-2^[42]^. In the case of M1, however, ZAP-S dominance appears to facilitate rather than block viral propagation.

To test the hypothesis that IRE1α protects cells from M1-induced ER stress-mediated death, we used both pharmacological inhibition and genetic knockdown strategies. Pre-treatment with the selective IRE1α inhibitor STF significantly enhanced M1 cytotoxicity at low MOIs in both T24 and UM-UC-3 cells. However, this enhancement was not observed at higher MOIs (1 or 10), possibly due to M1-induced IRE1α upregulation that occurs after infection, which may not be effectively blocked by transient pre-treatment. To address this limitation, we introduced siRNA to knock down IRE1α expression. Suppression of IRE1α in both T24 and UM-UC-3 bladder cancer cell lines resulted in a marked increase in M1-induced ER stress and apoptotic signaling. This was reflected by elevated levels of CHOP, enhanced phosphorylation of JNK, and cleavage of caspase-12. These results confirm that IRE1α serves as a cellular resistance factor that limits the oncolytic efficacy of M1 virus by facilitating viral protein degradation. Interestingly, while knockdown of IRE1α led to a noticeable increase in viral protein levels, subsequent experiments showed no significant change in viral titer or fluorescence signal. This indicates that the observed enhancement in M1-induced cell death is more likely due to reduced degradation of viral proteins and intensified ER stress, rather than increased viral replication. These results suggest that targeting IRE1α boosts the cytotoxic impact of M1 virus through a host-driven mechanism, without promoting viral spread, and may offer a safer and more controllable therapeutic strategy.

Several limitations of this study should be acknowledged. First, although we validated our findings using both *in vitro* and *in vivo* models, these preclinical settings may not fully recapitulate the tumor microenvironment and immune interactions present in human bladder cancer. Second, the number of PCBC samples and clinical datasets analyzed was limited. Although TCGA analysis suggested a trend toward poorer prognosis in patients with low IRE1α expression, the association did not reach statistical significance (*P* = 0.065), warranting validation in larger and independent cohorts. Finally, our use of a single xenograft model and immunodeficient mice limits the generalizability of the therapeutic synergy between M1 virus and IRE1α inhibition, particularly in the context of antitumor immunity. Future work should include immunocompetent models and clinical samples to better evaluate translational potential.

In conclusion, our study demonstrates that M1-induced ER stress, particularly via IRE1α signaling, plays a pivotal role in mediating selective oncolysis in bladder cancer. M1-sensitive tumors typically express low levels of IRE1α. Interestingly, M1 infection still activates IRE1α, which promotes viral protein degradation. When IRE1α is inhibited, viral protein accumulation increases, triggering stronger ER stress and apoptosis. These findings reveal a promising therapeutic window and provide a strong rationale for combining M1 virotherapy with ER stress modulation to improve efficacy and tumor selectivity in bladder cancer.

## DECLARATIONS

### Availability of data and materials

The data that support the findings of this study are available from the corresponding author upon reasonable request.

### Copyright

© The Author(s) 2025.
